# Did you choose appropriate tracer for retrograde tracing of retinal ganglion cells? The differences between cholera toxin subunit B and Fluorogold

**DOI:** 10.1371/journal.pone.0205133

**Published:** 2018-10-05

**Authors:** Fei Yao, Endong Zhang, Zhaolin Gao, Hongpei Ji, Mahmoud Marmouri, Xiaobo Xia

**Affiliations:** 1 Department of Ophthalmology, Xiangya Hospital, Central South University, Changsha, Hunan, China; 2 Department of Ophthalmology, The People’s Hospital of Guizhou Province, Guiyang, Guizhou, China; University of Florida, UNITED STATES

## Abstract

Cholera toxin subunit B (CTB) and Fluorogold(FG) are two widely utilized retrograde tracers to assess the number and function of retinal ganglion cells (RGCs). However, the relative advantages and disadvantages of these tracers remain unclear, which may lead to their inappropriate application. In this study, we compared these tracers by separately injecting the tracer into the superior Colliculi (SC) in rats, one or 2 weeks later, the rats were sacrificed, and their retinas, brains, and optic nerves were collected. From the first to second week, FG displayed a greater number of labeled RGCs and a larger diffusion area in the SC than CTB; The number of CTB labeled RGCs and the diffusion area of CTB in the SC increased significantly, but there was no distinction between FG; Furthermore, CTB exhibited more labeled RGC neurites and longer neurites than FG, but no difference was evident between the same trace; The optic nerves labeled using CTB were much clearer than those labeled using FG. In conclusion, both CTB and FG can be used for the retrograde labeling of RGCs in rats at 1 or 2 weeks. FG achieves retrograde labeling of a greater number of RGCs than CTB, whereas CTB better delineates the morphology of RGCs. Furthermore, CTB seems more suitable for retrograde labeling of some small, non-image forming nuclei in the brain to which certain RGC subtypes project their axons.

## Introduction

Retinal ganglion cells (RGCs) are the main projecting retinal neurons in the visual pathway. They project their axons directly to the brain to perform image-forming and non-image forming functions, and they are the only afferent neurons from the retina [1–4].

Numerous studies have demonstrated that diseases such as glaucomatous optic neuropathy [5–7], traumatic optic neuropathy [8–10], ischemic optic neuropathy [11–13], and retinal degeneration [14, 15] can lead directly or indirectly to RGC dysfunction or loss. RGCs are often monitored during the investigation of such diseases, especially when evaluating the effects of neuroprotective therapies [16–18]. Therefore, accurate and reliable methods of assessing the number and functional capacity of surviving RGCs are highly sought after. Techniques for evaluating RGC loss and dysfunction have been reviewed by Mead and Tomarev [19].

Retrograde tracing is a feasible and effective way to investigate RGCs via animal experimentation [19]. A retrograde tracer is injected at or close to the axon terminals of RGCs, which in higher primates mainly reside in the lateral geniculate nucleus (LGN) and Superior Colliculi (SC) [20, 21] and in rodents mainly reside in the SC [22, 23]. The axon terminals then take up the tracer via an active or passive mechanism and transport it from peripheral axons to the parent somata and dendrites by axonal flow [24, 25]. Later, RGCs containing tracers can be visualized using techniques specific to each tracer. Via this mechanism, RGCs alone can be stained in the retina [22]. In addition, because healthy axonal flow is essential for the retrograde labeling of RGCs, which is considered to involve active transport [25], any axonal dysfunction will directly influence the number of labeled RGCs [19, 26]. Therefore, retrograde tracing may be more sensitive to RGC dysfunction or loss.

Cholera toxin subunit B (CTB) and Fluorogold(FG) are retrograde tracers widely used in many laboratories [27–33]. CTB is a nontoxic subunit of the cholera toxin protein complex secreted by Vibrio cholerae that binds to cellular surfaces via the pentasaccharide chain of monosialotetrahexosylganglioside [34]. Subsequently, CTB is selectively taken up into the cytoplasm by adsorptive endocytosis [35]. In fact, CTB can also be used as an anterograde tracer [28, 35, 36], whereas FG can only be utilized as a retrograde tracer [27]. FG was first utilized as a retrograde fluorescent tracer by Schmued and Fallon [37]. The active constituent of FG is hydroxystilbamidine, a weak base that emits light upon excitation with ultraviolet radiation. The uptake mechanism of FG is passive incorporation [38].

The efficiencies of these tracers for the retrograde labeling of RGCs have been confirmed in previous studies [31, 39–41]. However, their relative advantages and disadvantages remain unclear, which may lead to their inappropriate application. In this study, we compared CTB and FG for the retrograde tracing of RGCs in rats with the aim of clarifying their specific features.

## Materials and methods

### Tracers

Alexa Fluor (AF) 555-conjugated CTB (AF 555-CTB; Molecular Probes, Eugene, OR, USA) dissolved in phosphate-buffered saline (PBS) at a concentration of 1% was used for CTB injection. FG (Fluorochrome, LLC, Denver, CO, USA) dissolved in 0.9% saline at a concentration of 4% was used for FG injection. Both solutions were stored at −20°C until use. In addition, a mixed solution for CTB + FG injection was prepared using isopyknic 1% CTB and 4% FG solutions.

### Animals

Twenty adult Sprague—Dawley (SD) rats (female; 200–250 g; 8 weeks old) purchased from Animal Laboratory Supplies (Xiangya School of Medicine, Central South University, Changsha, Hunan Province, China) were used in this study, which were equally divided into four groups according to postoperative survival time and the tracers injected into the SC (Table 1). Besides, in order to observe the CTB-labeled RGCs and FG-labeled RGCs in the same rat, additional six rats were injected with a mixed solution of CTB and FG and allowed to survive for 1 (n = 3 rats, 6 eyes) or 2 weeks (n = 3 rats, 6 eyes). All animals were housed in comfortable conditions with free access to food and water under a 12-hour light/12-hour dark cycle. All experimental procedures were reviewed and approved by the Animal Care and Use Committees of the Laboratory Animal Research Center at Xiangya Medical School of Central South University.

**Table 1:** Animal grouping.

Group	Tracer	Survival time (days)	Number of rats	Number of eyes
CTB-1w	1% CTB	7	5	10
CTB-2w	1% CTB	14	5	10
FG-1w	4% FG	7	5	10
FG-2w	4% FG	14	5	10

CTB, cholera toxin subunit B; CTB-1w, rats euthanized 1 week post-CTB injection; CTB-2w, rats euthanized 2 weeks post-CTB injection; FG, Fluorogold; FG-1w, rats euthanized 1 week post-FG injection; FG-2w, rats euthanized 2 weeks post-FG injection.

### Retrograde tracing

Animals were anesthetized with a mixed solution of 2% sodium pentobarbital (80 mg·kg^−1^) and xylazine (10 mg·kg^−1^) administered by intraperitoneal injection. Fully anesthetized animals with a regular respiratory rate and no pain reaction to a toe pinch were fixed in a stereotaxic instrument (RWD Life Science Co., Ltd., Shenzhen, Guangdong Province, China) using blunt earbars. Then, a surgical blade was used to make an incision to expose the bregma and posterior fontanelle. The skull surface localizations of the bilateral SC are 6 mm posterior to the bregma and 1.8 mm lateral to the midline. Two small holes (2.5 mm in diameter) were made using a mini drill on both sides of the midline. A 5-μl Hamilton syringe was vertically inserted to a depth of 4 mm, and used to slowly pressure-inject 0.5 μl of tracer into the ambilateral SC. Placement of the syringe in the target zone and tracer injection took 10 and 3 min, respectively. Following the completion of two-site injection, 4–0 nonabsorbable surgical threads (Ethicon, Inc., Somerville, NJ, USA) were used to suture the incision. To reduce the risk of infection and provide pain relief, an antibiotic and an analgesic were added to the rats’ drinking water. During the whole procedure, 0.9% saline was used to keep the rats’ eyes hydrated, and a heating lamp was used to keep the rats warm.

### Tissue preparation

One and 2 weeks later, the rats were re-anesthetized and perfused transcardially with PBS followed by 4% paraformaldehyde. Then, microscissors and fine forceps were used to enucleate each intact eye and carefully remove the cornea, lens, and vitreous humor. Simultaneously, a small incision was made as a marker on the inferior border of the eyecup to distinguish retinal direction. Next, the eyecups were fixed in 4% paraformaldehyde for 1 h at room temperature before detachment of the retinas. To stretch the retinas flat, they were placed with ganglion cell layers facing upward on superfrost plus microscope slides and incised with four 3 mm-long cuts at their superior, inferior, temporal, and nasal sides according to the marker incision made previously. Finally, the retinas were cleaned using a soft brush, covered with anti-fade aqueous mounting medium, and coverslipped. All detailed procedures were conducted using a stereomicroscope. The brains and intact optic nerves were fixed in 4% paraformaldehyde for 24 h, and dehydrated in 30% phosphate-buffered sucrose solution at 4°C until the tissues sank. To observe the injection sites and morphology of the optic nerves, the dewatered brains and optic nerves were cut into slices of different thicknesses (30 μm for brain and 15 μm for optic nerves) using a freezing sliding microtome (CM3050 S; Leica Microsystems GmbH, Wetzlar, Germany).

### Fluorescence microscopy and image analysis

All flattened retinas and tissue sections were examined using a fluorescence microscope (DM5000 B; Leica Microsystems GmbH). CTB was examined using Filter N21 (band pass 515 nm, long pass 560 nm) and FG was examined using Filter A (band pass 340 nm, long pass 380 nm). To capture the whole retina, 12 pictures of each retina were taken at high magnification (313.28 × 235.89 μm^2^) at distances of 0.85, 2.26, and 3.68 mm (approximately 1/6, 1/2, and 5/6 the radius of the retina) from the optic disc in the superonasal, inferonasal, superotemporal and inferotemporal quadrants. Brain and optic nerve sections were also photographed. All pictures were analyzed using Image-Pro Plus 6.0 (Media Cybernetics, Inc., Bethesda, MD, USA). To calculate the mean density of labeled RGCs in each retina, a manual counter was used to count the labeled cells in each picture. An atlas of the rat brain (*The Rat Brain in Stereotaxic Coordinates*; Elsevier Inc., 2007) was used to identify the injection sites.

### Statistical analysis

In order to account for the correlation between the right and left eyes and analyze the statistical differences in the mean density of RGCs (MD-RGCs), mean amount of neurites of labeled RGCs (MAN-RGCs), mean length of neurites of labeled RGCs (MLN-RGCs), and diffusion area in the SC (DA-SC) between the rats euthanized 1 week post-CTB injection (CTB-1w; *n* = 5 rats, 10 eyes), rats euthanized 2 weeks post-CTB injection (CTB-2w; *n* = 5 rats, 10 eyes), rats euthanized 1 week post-FG injection (FG-1w; *n* = 5 rats, 10 eyes), and rats euthanized 2 weeks post-FG injection (FG-2w; *n* = 5 rats, 10 eyes),two-way analysis of variance (ANOVA) was used. In addition, Tukey test was used as post hoc test to perform multiple comparisons between the 4 groups. All data were presented as the mean ± standard error of the mean, and statistical significance was set at p <0.05.

## Results

### Mean density of labeled retinal ganglion cells

**Fig 1:**
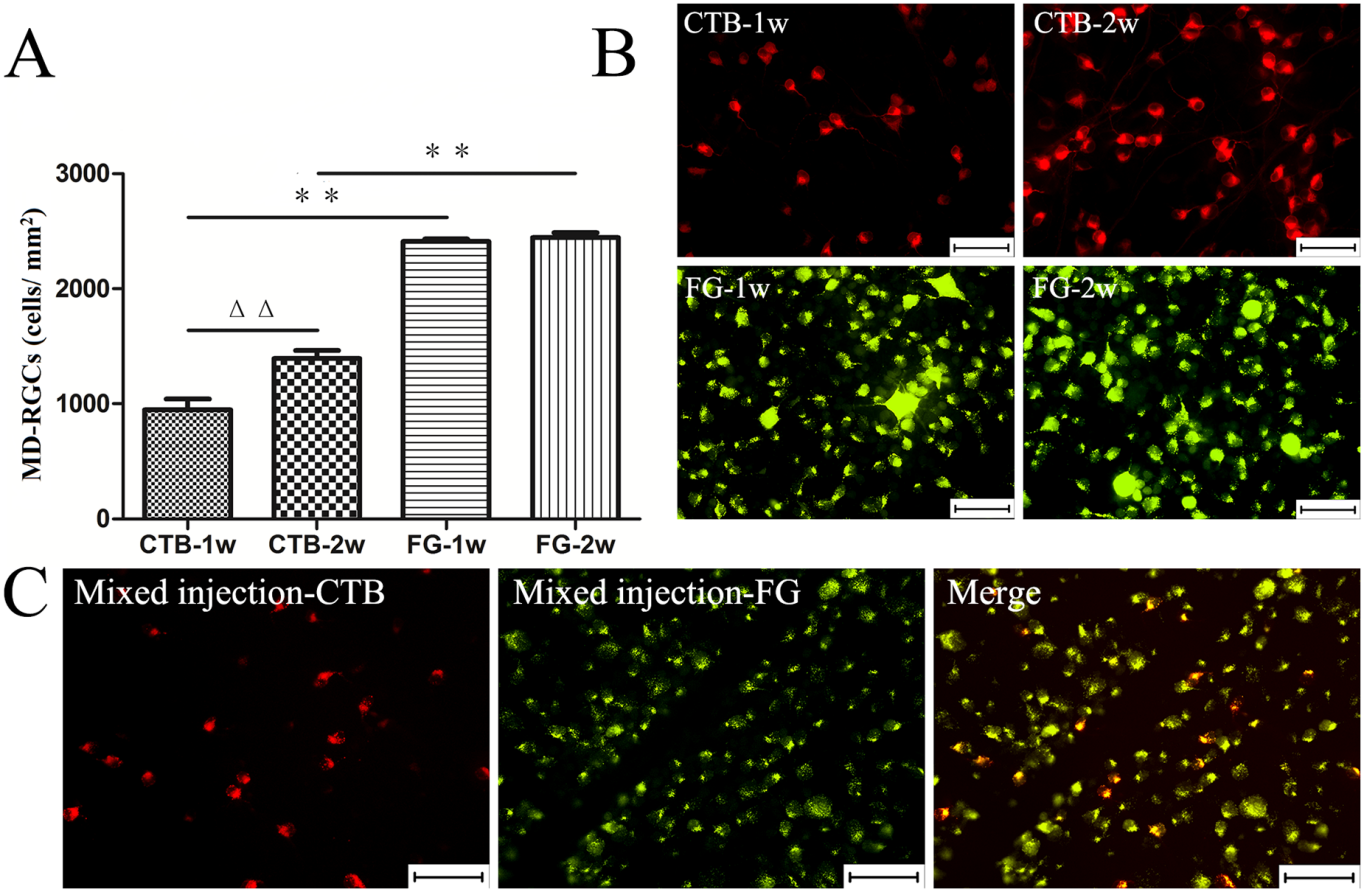
The mean density of retinal ganglion cells (MD-RGCs) retrograde labeled by cholera toxin subunit B (CTB) and Fluorogold(FG) at 1 week and 2 weeks in rats. (A) MD-RGCs in CTB-1w (rats euthanized 1 week post-CTB injection) and CTB-2w (rats euthanized 2 weeks post-CTB injection) were less than those in FG-1w (rats euthanized 1 week post-FG injection) and FG-2w (rats euthanized 2 weeks post-FG injection) (p <0.001), MD-RGCs in CTB-2w was greater than that in CTB-1w (p <0.001), but there was no distinction between the FG-1w and FG-2w (p = 0.982). (B) Representative photomicrographs taken at approximately 5/6 retinal radius in the superonasal quadrant showing retinal ganglion cells (RGCs) labeled by CTB and FG at 1week and 2 weeks. (C) In the rat euthanized 2 week post-mixed injection (CTB+FG), the number of FG-labeled RGCs far exceeded that of CTB-labeled RGCs in an identical visual field, the pictures were taken at approximately 1/2 retinal radius in the superotemporal quadrant. ^ΔΔ^Two-way analysis of variance (ANOVA), p <0.001; **two-way ANOVA, p <0.001. Scale bar = 50 μm.

The uptake efficacy of CTB and FG was evident in the MD-RGCs (Table 2 and Fig 1). All groups exhibited detectable staining after tracer injection, but the MD-RGCs differed significantly between the CTB and FG Groups. The FG-1w and FG-2w Groups displayed a greater number of labeled RGCs than the CTB-1w (p <0.001) and CTB-2w (p <0.001) Groups (Fig 1A and 1B). This difference was also demonstrated by the CTB + FG-injected rats (Fig 1C): the number of FG-labeled RGCs far exceeded that of CTB-labeled RGCs in an identical visual field. Regarding the MD-RGCs of the same tracer at different times, there was no distinction between the FG-1w and FG-2w Groups (p = 0.982), but the CTB-2w Group showed a greater MD-RGCs than the CTB-1w Group (p <0.001). In addition, no difference between the right and left eyes (p = 0.287) and no interaction between the groups and eyes (p = 0.114) were found in this study.

**Table 2:** Results of retrograde tracing using cholera toxin subunit B (CTB) and Fluorogold(FG) at 1 week and 2 weeks in rats.

	CTB-1w (*n* = 10 eyes)	CTB-2w (*n* = 10 eyes)	FG-1w (*n* = 10 eyes)	FG-2w (*n* = 10 eyes)
MD-RGCs (cells/mm^2^)	948 ± 93	1394 ± 70^ΔΔ^	2411 ± 21**	2443 ± 45**
MAN-RGCs	1.81 ± 0.05	1.88 ± 0.06	1.03 ± 0.05**	1.09 ± 0.04**
MLN-RGCs (μm)	46.7 ± 2.9	54 ± 2.5	20.5 ± 1.4**	20.3 ± 0.8**
DA-SC (mm^2^)	0.405 ± 0.017	0.955 ± 0.028^ΔΔ^	2.967 ± 0.166**	3.180 ± 0.201**

Means ± standard error of the mean unless otherwise stated.

CTB-1w, rats euthanized 1 week post-CTB injection; CTB-2w, rats euthanized 2 weeks post-CTB injection; DA-SC, diffusion area in the superior colliculus; FG-1w, rats euthanized 1 week post-FG injection; FG-2w, rats euthanized 2 weeks post-FG injection; MAN-RGCs, mean amount of neurites of labeled retinal ganglion cells; MD-RGCs, mean density of labeled retinal ganglion cells; MLN-RGCs, mean length of neurites of labeled retinal ganglion cells.

^ΔΔ^Two-way analysis of variance (ANOVA), p <0.001(same tracers compared at different times);

**two-way ANOVA, p <0.001 (CTB and FG compared at the same time).

### Morphology of labeled retinal ganglion cells

**Fig 2:**
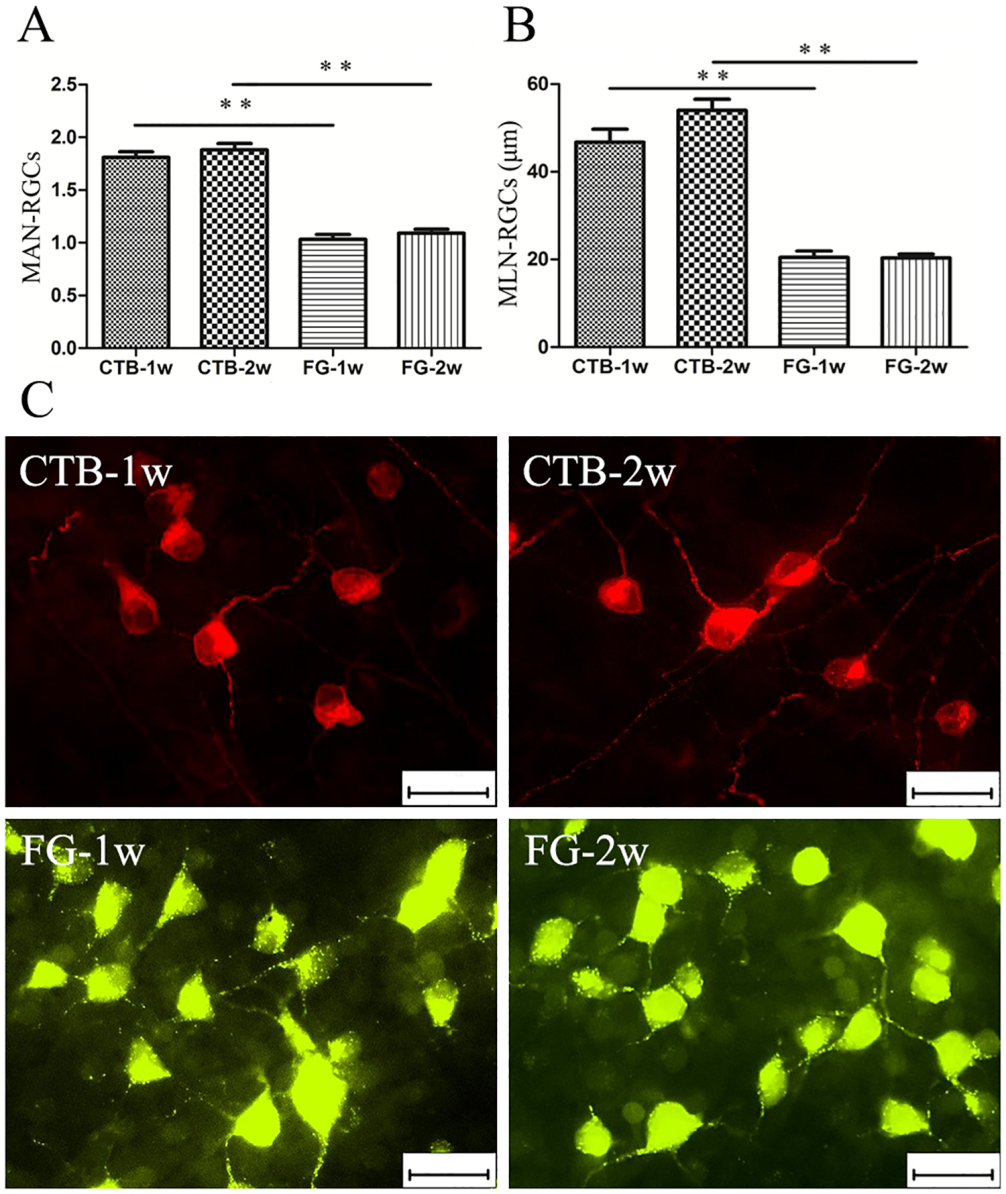
The morphology of cholera toxin subunit B (CTB)-retrograde labeled and Fluorogold(FG)-retrograde labeled retinal ganglion cells (RGCs) at 1 week and 2 weeks in rats. (A) The mean amount of neurites of labeled RGCs (MAN-RGCs) in CTB-1w (rats euthanized 1 week post-CTB injection) and CTB-2w (rats euthanized 2 weeks post-CTB injection) were more than those in FG-1w (rats euthanized 1 week post-FG injection) and FG-2w (rats euthanized 2 weeks post-FG injection) (p <0.001), but there was no distinction between the CTB-1w and CTB-2w (p = 0.749) or between the FG-1w and FG-2w (p = 0.842). (B) In terms of the mean length of neurites of labeled RGCs (MLN-RGCs), The CTB-1w and CTB-2w exhibited longer labeled RGC neurites than the FG-1w and FG-2w (p <0.001), but no difference was evident between the CTB-1w and CTB-2w (p = 0.111) or between the FG-1w and FG-2w (p = 1.0). (C) Representative photomicrographs taken at approximately 5/6 retinal radius in the superonasal quadrant showing the neurites of RGCs labeled by CTB and FG at 1 week and 2 weeks. **Two-way analysis of variance (ANOVA), p <0.001. Scale bar = 25 μm.

To further study the properties of CTB and FG, 10 retinal images photographed at 5/6 the retinal radius (3.68 mm from the optic disc) were randomly selected from each group to analyze the MAN-RGCs and MLN-RGCs (Table 2 and Fig 2). The MAN-RGCs in the FG Groups were less than those in the CTB Groups at both 1 (p <0.001) and 2 (p <0.001) weeks, but no differences were evident between the CTB-1w and CTB-2w Groups (p = 0.749) or between the FG-1w and FG-2w Groups (p = 0.842; Fig 2A). In terms of MLN-RGCs, there was no distinction between the same tracer at 1 and 2 weeks: the CTB-1w Group was similar to the CTB-2w Group (p = 0.111), and the FG-1w Group resembled the FG-2w Group (p = 1.0). However, CTB-labeled RGC neurites were approximately two times longer than FG-labeled RGC neurites (Fig 2B). In addition, as shown in Fig 2C, round or ovoid somata labeled by CTB and FG both exhibited a clear plasma membrane, with bright staining of the cytoplasm and no staining of the nuclei, but the cytoplasm stained by FG had a more coarse and granular appearance than that stained by CTB. In MAN-RGCs or MLN-RGCs, there was no difference between the right and left eyes (p = 0.827 in MAN-RGCs, p = 0.934 in MLN-RGCs), and no interaction between the groups and eyes (p = 0.222 in MAN-RGCs, p = 0.957 in MLN-RGCs).

### Diffusion area in the superior Colliculi

**Fig 3:**
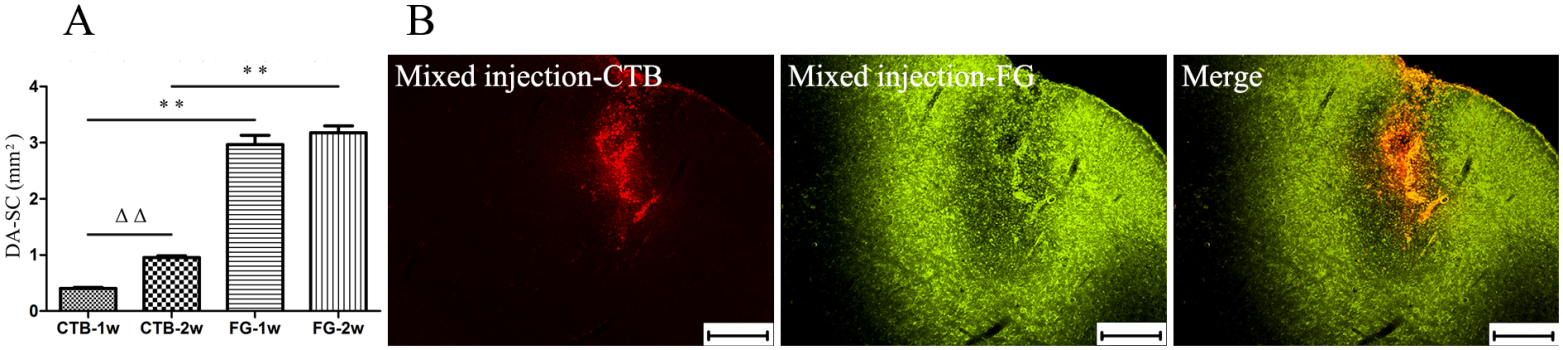
The diffusion area of cholera toxin subunit B (CTB) and Fluorogold(FG) in the superior colliculus (SC) at 1 week and 2 weeks in rats. (A) The diffusion area of tracers in the SC (DA-SC) in FG-1w (rats euthanized 1 week post-FG injection) and FG-2w (rats euthanized 2 weeks post-FG injection) were much larger than those in CTB-1w (rats euthanized 1 week post-CTB injection) and CTB-2w (rats euthanized 2 weeks post-CTB injection) (p <0.001), CTB-2w displayed a larger DA-SC than CTB-1w (p <0.005), but the DA-SC in FG-1w and FG-2w were similar (p = 0.513). (B) In an identical visual field of SC of a rat euthanized 2 week post-mixed injection (CTB+FG), the SC stained by CTB was focused and small, whereas the area stained by FG was diffuse and wide. ^ΔΔ^Two-way analysis of variance (ANOVA), p <0.001; **two-way ANOVA, p <0.001. Scale bar = 200 μm.

To verify the injection sites and compare the diffusion characteristics of CTB and FG, slices of the bilateral SC were photographed and studied. All injection sites were first confirmed to be in the SC according to the atlas of the rat brain, and then Image-Pro Plus 6.0 was used to measure the DA-SC of each injection site where the needle passage was most obvious. As shown in Table 2 and Fig 3A, the DA-SC in the FG Groups was much larger than that in the CTB Groups, both at 1 (p <0.001) and 2 (p <0.001) weeks. This remarkable difference was also evident in the mixed-injection rats (Fig 3B): the SC stained by CTB was focused and small, whereas the area stained by FG was diffuse and wide. An increasing trend in the DA-SC was found in the CTB Groups (p <0.005) from 1 to 2 weeks, but not in the FG Groups (p = 0.513). Besides, no difference between the right and left SC (p = 0.885) and no interaction between the groups and bilateral SC (p = 0.748) were found.

### Characteristics of labeled optic nerves

**Fig 4:**
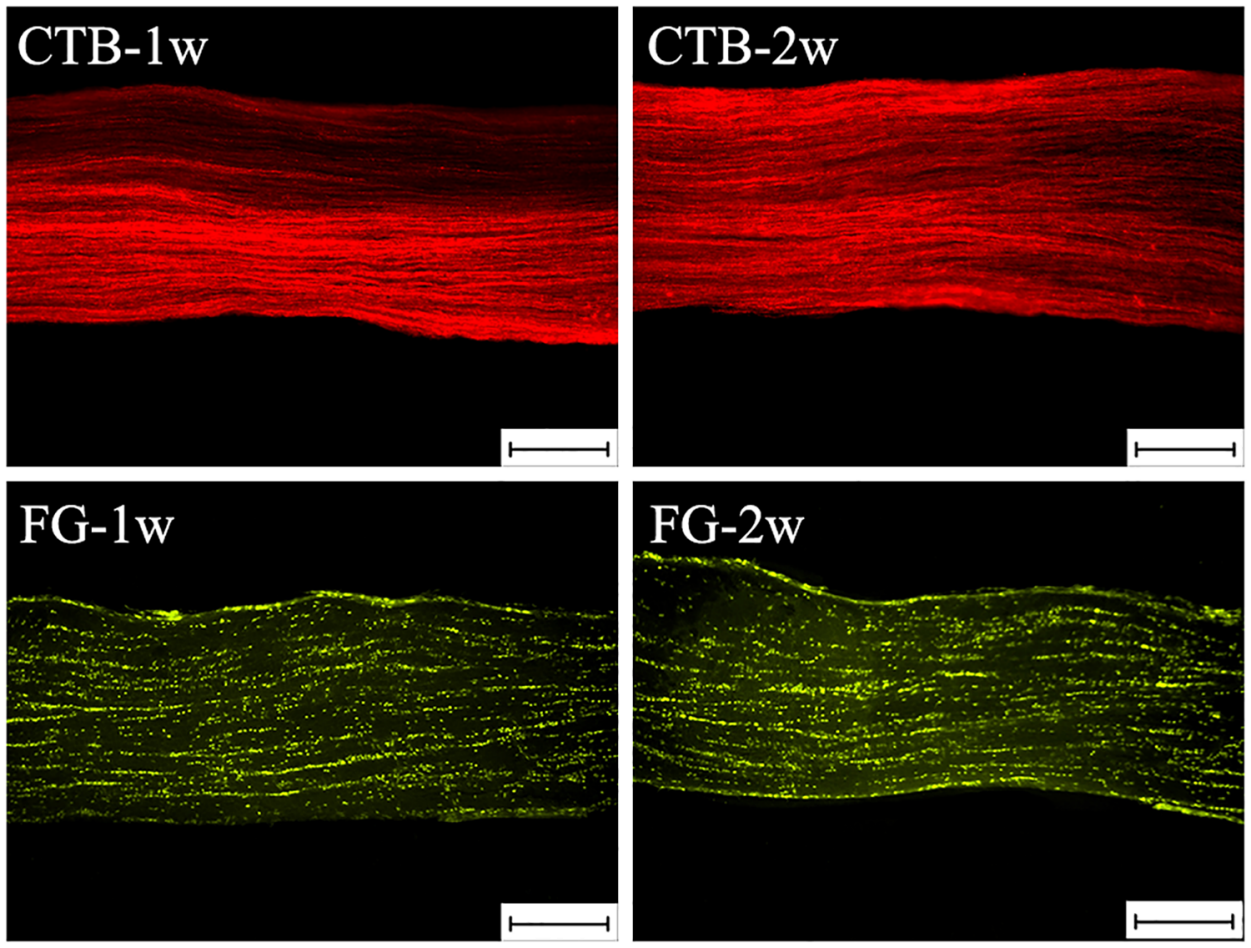
The characteristics of optic nerves retrograde labeled using cholera toxin subunit B (CTB) and Fluorogold(FG) at 1 week and 2 weeks in rats. The optic nerves labeled using CTB were much clearer than those labeled using FG. CTB-1w, rats euthanized 1 week post-CTB injection; CTB-2w, rats euthanized 2 weeks post-CTB injection; FG-1w, rats euthanized 1 week post-FG injection; FG-2w, rats euthanized 2 weeks post-FG injection. Scale bar = 200 μm.

The characteristics of labeled optic nerves were also examined (Fig 4). The optic nerves labeled by FG showed discrete linear fluorescence without detectable dye leakage. The axons were segmentally stained and presented a granular appearance; in fact, most axons were not clearly labeled. In contrast, the optic nerves labeled by CTB were much more distinct: the axons displayed complete linear structure. In addition, the number of CTB-labeled axons was much greater than that of FG-labeled axons at 1 and 2 weeks. A slight increase was found in the CTB Groups from 1 to 2 weeks, but no obvious difference was found between the FG-1w and FG-2w Groups.

## Discussion

Our study first comprehensively compared the differences between the two commonly used retrograde tracers, CTB and FG, in retrograde labeling of RGCs. In previous studies, many investigators prefer CTB for the retrograde labeling of RGCs because of its high efficiency: a relatively low concentration and low dose of CTB could achieve a satisfactory labeling effect [35]. While Some researchers prefer FG for the retrograde labeling of RGCs because of its intense fluorescence, extensive filling of dendrites, high resistance to fading, and wide latitude of survival time [27, 37, 38]. However, up to now, no study has expressly indicated which tracer is more suitable for retrograde labeling RGCs.

In order to elucidate the specific features of CTB and FG, we not only compared their differences in labeling the mean density of RGCs, but also compared their diffusion ability in SC and their morphological differences in labeling the neurites of RGCs and optic nerve. In terms of survival time, there is no benchmark of CTB: from 4 days to 4 weeks is feasible [35]. The axonal transport rate of CTB is approximately 160 mm/day in rats [25]. The time taken by FG to travel from the SC to the RGC somata is reportedly within 1 week in SD rats [22]. Also, it is recommended that the survival time of FG should be kept short because evidence has shown that, with long-term use, FG can diffuse into amacrine cells and microglial cells and disrupt neuronal function [42–45]. Accordingly, we chose 1 and 2 weeks as the time points to observe the results of retrograde tracing.

Our results demonstrate that FG can retrogradely label RGCs more completely than CTB at both 1 and 2 weeks. The mean densities of FG-labeled RGCs observed in our experiments (2411 ± 21 cells/mm^2^ at 1 week and 2443 ± 45 cells/mm^2^ at 2 weeks) are consistent with that reported in Chien’s study (2359 ± 423 cells/mm^2^), Chang’s study (2430 ± 120 cells/mm^2^), Lindqvist’s study (2511 ± 20 cells /mm^2^), and Jehle’s study (2456 ± 141 cells/mm^2^) [31, 46–48], but differ from that presented in studies by Wang et al (1802.00 ± 115.40 cells/mm^2^) [49], a possible explanation for this discrepancy is that the fluorescent images were obtained at different distances from the optic disc. The densities of CTB-labeled RGCs in our study (948 ± 93 cells/mm^2^ at 1 week and 1394 ± 70 cells/mm^2^ at 2 weeks) also differ from that reported in a study by Choe et al (about 2000 cells/mm^2^) [41], what acounts for the differences is that they injected CTB into SC at -4.5, -4.25, -4.0, and -3.75 mm depth from the skull surface to retrograde label RGCs, while we injected CTB only at a single -4.0 mm depth. Despite these slight discrepancies in RGC density, all of these studies confirm our finding that FG labeled significantly more RGCs than CTB. Thus, FG shows superiority over CTB in terms of labeling the greatest number of RGCs, meaning that FG is more suitable for studies that aim to observe all RGCs, such as studies on RGC apoptosis or protection [16–18, 50].

To further analyze the differences between CTB and FG, we measured the diffusion areas of each tracer in the SC, and found that FG diffused more easily than CTB: the diffusion areas of FG were much larger than those of CTB at 1 and 2 weeks. Simultaneously, an interesting phenomenon was noted in the CTB Groups, but not in the FG Groups: the diffusion area of CTB markedly increased from 1 to 2 weeks. A reasonable interpretation of this finding is that the diffusion efficiency of CTB in the SC is lower than that of FG: whereas FG reached nearly maximum diffusion at 1 week, CTB was still diffusing. In Choe’s and Abbott’s studies [25, 41], they found that injecting CTB at four depth from the skull surface could usually diffuse well throughout the SC, which is consistent with our conclusion that the diffusion ability of CTB in SC is relatively weak, but they didn’t investigate the features of FG in their studies. As previously described, RGCs ingest tracers from the SC [23]: with a larger area of the SC stained, a greater number of RGCs can be labeled by tracers, which may also explain the distinction between the CTB-1w and CTB-2w Groups in RGC density. This result suggests that CTB is more suitable for the retrograde tracing of RGC subtypes that project their axons to small, non-image forming nuclei in the brain, such as the suprachiasmatic nucleus [51], dorsal raphe nucleus [52], and caudal periaqueductal gray [53], because CTB can be injected into small areas without staining the surrounding nuclei.

Regarding morphology, both CTB and FG intensely stained RGCs, with bright staining of the cytoplasm and no staining of the nuclei. The labeled somata exhibited a granular appearance, consistent with previous studies [27, 35, 54]. The morphologic results demonstrated that CTB displayed RGC neurites, including axons, more clearly than FG, although the specific reason for this remains unclear. We conjecture that it is due to the tracers’ individual natures; indeed, CTB is suitable for use both as a retrograde and an anterograde tracer [28, 35, 36], whereas FG can only be utilized as a retrograde tracer [27]. This means that CTB may be transported from cell bodies to cell processes and thereby stain neurites more clearly. No matter the specific reason, CTB is more suitable for studies that intend to investigate the morphology of RGCs, especially the morphology of RGC axons, such as assessments of axonal damage and regrowth [55].

In conclusion, we compared two commonly used retrograde tracers, CTB and FG, for the labeling of RGCs in rats at 1 and 2 weeks. Our results demonstrate that both CTB and FG can successfully label the RGCs of rats at 1 and 2 weeks. FG labels more RGCs than CTB, whereas CTB better outlines the morphology of RGCs. Furthermore, CTB facilitates the retrograde labeling of some small, non-image forming nuclei in the brain to which some RGC subtypes project their axons.

## References

[1] Ramsey DJ, Ramsey KM, Vavvas DG. Genetic advances in ophthalmology: the role of melanopsin-expressing, intrinsically photosensitive retinal ganglion cells in the circadian organization of the visual system. Semin Ophthalmol. 2013;28(5–6):406–21. 10.3109/08820538.2013.825294 . 24010846

[2] Schmidt TM, Chen SK, Hattar S. Intrinsically photosensitive retinal ganglion cells: many subtypes, diverse functions. Trends Neurosci. 2011;34(11):572–80. 10.1016/j.tins.2011.07.001 . 21816493 PMC3200463

[3] Sanes JR, Masland RH. The types of retinal ganglion cells: current status and implications for neuronal classification. Annu Rev Neurosci. 2015;38:221–46. 10.1146/annurev-neuro-071714-034120 . 25897874

[4] Isenmann S, Kretz A, Cellerino A. Molecular determinants of retinal ganglion cell development, survival, and regeneration. Prog Retin Eye Res. 2003;22(4):483–543. . 12742393 10.1016/s1350-9462(03)00027-2

[5] Dang YL, Waxman S, Wang C, Parikh HA, Bussel II, Loewen RT, et al Rapid learning curve assessment in an ex vivo training system for microincisional glaucoma surgery. Sci Rep-Uk. 2017;7 10.1038/s41598-017-01815-z 28487512 PMC5431621

[6] Levkovitch-Verbin H. Retinal ganglion cell apoptotic pathway in glaucoma: Initiating and downstream mechanisms. Prog Brain Res. 2015;220:37–57. 10.1016/bs.pbr.2015.05.005 . 26497784

[7] Li HB, You QS, Xu LX, Sun LX, Abdul Majid AS, Xia XB, et al Long Non-Coding RNA-MALAT1 Mediates Retinal Ganglion Cell Apoptosis Through the PI3K/Akt Signaling Pathway in Rats with Glaucoma. Cell Physiol Biochem. 2017;43(5):2117–32. 10.1159/000484231 . 29065394

[8] Wilhelm H. [Traumatic optic neuropathy]. Laryngorhinootologie. 2009;88(3):194–203; quiz 4–7. 10.1055/s-0029-1192010 . 19247896

[9] Jiang B, Zhang P, Zhou D, Zhang J, Xu X, Tang L. Intravitreal transplantation of human umbilical cord blood stem cells protects rats from traumatic optic neuropathy. Plos One. 2013;8(8):e69938 10.1371/journal.pone.0069938 . 23940534 PMC3734232

[10] Al-Zubidi N, Ansari W, Fung SH, Lee AG. Diffusion tensor imaging in traumatic optic tract syndrome. J Neuroophthalmol. 2014;34(1):95–8. 10.1097/WNO.0000000000000069 . 24162259

[11] Hayreh SS. Ischemic optic neuropathy. Prog Retin Eye Res. 2009;28(1):34–62. 10.1016/j.preteyeres.2008.11.002 . 19063989

[12] Shao X, He Z, Tang L, Gao L. Tacrolimus-associated ischemic optic neuropathy and posterior reversible encephalopathy syndrome after small bowel transplantation. Transplantation. 2012;94(9):e58–60. 10.1097/TP.0b013e31826dde21 . 23128973

[13] Pereira LS, Avila MP, Salustiano LX, Paula AC, Arnhold E, McCulley TJ. Intravitreal Triamcinolone Acetonide Injection in a Rodent Model of Anterior Ischemic Optic Neuropathy. J Neuroophthalmol. 2018 10.1097/WNO.0000000000000639 . 29521709

[14] Garcia-Ayuso D, Di Pierdomenico J, Esquiva G, Nadal-Nicolas FM, Pinilla I, Cuenca N, et al Inherited Photoreceptor Degeneration Causes the Death of Melanopsin-Positive Retinal Ganglion Cells and Increases Their Coexpression of Brn3a. Invest Ophthalmol Vis Sci. 2015;56(8):4592–604. 10.1167/iovs.15-16808 . 26200499

[15] Garcia-Ayuso D, Galindo-Romero C, Di Pierdomenico J, Vidal-Sanz M, Agudo-Barriuso M, Villegas Perez MP. Light-induced retinal degeneration causes a transient downregulation of melanopsin in the rat retina. Exp Eye Res. 2017;161:10–6. 10.1016/j.exer.2017.05.010 . 28552384

[16] Jiang W, Tang L, Zeng J, Chen B. Adeno-associated virus mediated SOD gene therapy protects the retinal ganglion cells from chronic intraocular pressure elevation induced injury via attenuating oxidative stress and improving mitochondrial dysfunction in a rat model. Am J Transl Res. 2016;8(2):799–810. . 27158370 PMC4846927

[17] Aksar AT, Yuksel N, Gok M, Cekmen M, Caglar Y. Neuroprotective effect of edaravone in experimental glaucoma model in rats: a immunofluorescence and biochemical analysis. Int J Ophthalmol. 2015;8(2):239–44. 10.3980/j.issn.2222-3959.2015.02.05 . 25938034 PMC4413568

[18] Wostyn P, Killer HE, De Deyn PP. Glymphatic stasis at the site of the lamina cribrosa as a potential mechanism underlying open-angle glaucoma. Clin Exp Ophthalmol. 2017;45(5):539–47. 10.1111/ceo.12915 . 28129671

[19] Mead B, Tomarev S. Evaluating retinal ganglion cell loss and dysfunction. Exp Eye Res. 2016;151:96–106. 10.1016/j.exer.2016.08.006 . 27523467 PMC5045805

[20] Jeffries AM, Killian NJ, Pezaris JS. Mapping the primate lateral geniculate nucleus: a review of experiments and methods. J Physiol Paris. 2014;108(1):3–10. 10.1016/j.jphysparis.2013.10.001 . 24270042 PMC5446894

[21] Perry VH, Cowey A. Retinal ganglion cells that project to the superior colliculus and pretectum in the macaque monkey. Neuroscience. 1984;12(4):1125–37. . 6483194 10.1016/0306-4522(84)90007-1

[22] Salinas-Navarro M, Mayor-Torroglosa S, Jimenez-Lopez M, Aviles-Trigueros M, Holmes TM, Lund RD, et al A computerized analysis of the entire retinal ganglion cell population and its spatial distribution in adult rats. Vision Res. 2009;49(1):115–26. 10.1016/j.visres.2008.09.029 . 18952118

[23] Wang L, Sarnaik R, Rangarajan K, Liu X, Cang J. Visual receptive field properties of neurons in the superficial superior colliculus of the mouse. J Neurosci. 2010;30(49):16573–84. 10.1523/JNEUROSCI.3305-10.2010 . 21147997 PMC3073584

[24] Reynolds AJ, Bartlett SE, Hendry IA. Molecular mechanisms regulating the retrograde axonal transport of neurotrophins. Brain Res Brain Res Rev. 2000;33(2–3):169–78. . 11011064 10.1016/s0165-0173(00)00028-x

[25] Abbott CJ, Choe TE, Lusardi TA, Burgoyne CF, Wang L, Fortune B. Imaging axonal transport in the rat visual pathway. Biomed Opt Express. 2013;4(2):364–86. 10.1364/BOE.4.000364 . 23412846 PMC3567722

[26] Vidal-Sanz M, Valiente-Soriano FJ, Ortin-Martinez A, Nadal-Nicolas FM, Jimenez-Lopez M, Salinas-Navarro M, et al Retinal neurodegeneration in experimental glaucoma. Prog Brain Res. 2015;220:1–35. 10.1016/bs.pbr.2015.04.008 . 26497783

[27] Kobbert C, Apps R, Bechmann I, Lanciego JL, Mey J, Thanos S. Current concepts in neuroanatomical tracing. Prog Neurobiol. 2000;62(4):327–51. . 10856608 10.1016/s0301-0082(00)00019-8

[28] de Sousa TB, de Santana MA, Silva Ade M, Guzen FP, Oliveira FG, Cavalcante JC, et al Mediodorsal thalamic nucleus receives a direct retinal input in marmoset monkey (Callithrix jacchus): a subunit B cholera toxin study. Ann Anat. 2013;195(1):32–8. 10.1016/j.aanat.2012.04.005 . 22726524

[29] Krabichler Q, Vega-Zuniga T, Carrasco D, Fernandez M, Gutierrez-Ibanez C, Marin G, et al The centrifugal visual system of a palaeognathous bird, the Chilean Tinamou (Nothoprocta perdicaria). Journal of Comparative Neurology. 2017;525(11):2514–34. 10.1002/cne.24195 28256705

[30] Wang RB, Wu JC, Chen ZL, Xia FZ, Sun QL, Liu L. Postconditioning with inhaled hydrogen promotes survival of retinal ganglion cells in a rat model of retinal ischemia/reperfusion injury. Brain Research. 2016;1632:82–90. 10.1016/j.brainres.2015.12.015 26705611

[31] Chien JY, Sheu JH, Wen ZH, Tsai RK, Huang SP. Neuroprotective effect of 4-(Phenylsulfanyl)butan-2-one on optic nerve crush model in rats. Exp Eye Res. 2016;143:148–57. 10.1016/j.exer.2015.10.004 . 26472213

[32] Nadal-Nicolas FM, Salinas-Navarro M, Vidal-Sanz M, Agudo-Barriuso M. Two methods to trace retinal ganglion cells with fluorogold: from the intact optic nerve or by stereotactic injection into the optic tract. Exp Eye Res. 2015;131:12–9. 10.1016/j.exer.2014.12.005 . 25482219

[33] Zhao T, Li Y, Tang L, Li Y, Fan F, Jiang B. Protective effects of human umbilical cord blood stem cell intravitreal transplantation against optic nerve injury in rats. Graefes Arch Clin Exp Ophthalmol. 2011;249(7):1021–8. 10.1007/s00417-011-1635-7 . 21360302

[34] Wernick NL, Chinnapen DJ, Cho JA, Lencer WI. Cholera toxin: an intracellular journey into the cytosol by way of the endoplasmic reticulum. Toxins (Basel). 2010;2(3):310–25. 10.3390/toxins2030310 . 22069586 PMC3153193

[35] Lanciego JL, Wouterlood FG. A half century of experimental neuroanatomical tracing. Journal of Chemical Neuroanatomy. 2011;42(3):157–83. 10.1016/j.jchemneu.2011.07.001 21782932

[36] Scalia F, Rasweiler JJt, Danias J. Retinal projections in the short-tailed fruit bat, Carollia perspicillata, as studied using the axonal transport of cholera toxin B subunit: Comparison with mouse. J Comp Neurol. 2015;523(12):1756–91. 10.1002/cne.23723 . 25503714

[37] Schmued LC, Fallon JH. Fluoro-Gold: a new fluorescent retrograde axonal tracer with numerous unique properties. Brain Res. 1986;377(1):147–54. . 2425899 10.1016/0006-8993(86)91199-6

[38] Wessendorf MW. Fluoro-Gold: composition, and mechanism of uptake. Brain Res. 1991;553(1):135–48. . 1933270 10.1016/0006-8993(91)90241-m

[39] Marangoz D, Guzel E, Eyuboglu S, Gumusel A, Seckin I, Ciftci F, et al Comparison of the neuroprotective effects of brimonidine tartrate and melatonin on retinal ganglion cells. Int Ophthalmol. 2017 10.1007/s10792-017-0768-z . 29159432

[40] Abbott CJ, Choe TE, Lusardi TA, Burgoyne CF, Wang L, Fortune B. Evaluation of Retinal Nerve Fiber Layer Thickness and Axonal Transport 1 and 2 Weeks After 8 Hours of Acute Intraocular Pressure Elevation in Rats. Invest Ophth Vis Sci. 2014;55(2):674–87. 10.1167/iovs.13-12811 24398096 PMC3915863

[41] Choe TE, Abbott CJ, Piper C, Wang L, Fortune B. Comparison of Longitudinal In Vivo Measurements of Retinal Nerve Fiber Layer Thickness and Retinal Ganglion Cell Density after Optic Nerve Transection in Rat. Plos One. 2014;9(11). 10.1371/journal.pone.0113011 25393294 PMC4231142

[42] Abdel-Majid RM, Archibald ML, Tremblay F, Baldridge WH. Tracer coupling of neurons in the rat retina inner nuclear layer labeled by Fluorogold. Brain Res. 2005;1063(2):114–20. 10.1016/j.brainres.2005.09.046 . 16263096

[43] Hu W, Liu D, Zhang Y, Shen Z, Gu T, Gu X, et al Neurological function following intra-neural injection of fluorescent neuronal tracers in rats. Neural Regen Res. 2013;8(14):1253–61. 10.3969/j.issn.1673-5374.2013.14.001 . 25206419 PMC4107650

[44] Nadal-Nicolas FM, Jimenez-Lopez M, Salinas-Navarro M, Sobrado-Calvo P, Vidal-Sanz M, Agudo-Barriuso M. Microglial dynamics after axotomy-induced retinal ganglion cell death. J Neuroinflammation. 2017;14(1):218 10.1186/s12974-017-0982-7 . 29121969 PMC5679427

[45] Gomez-Ramirez AM, Villegas-Perez MP, Miralles de Imperial J, Salvador-Silva M, Vidal-Sanz M. Effects of intramuscular injection of botulinum toxin and doxorubicin on the survival of abducens motoneurons. Invest Ophthalmol Vis Sci. 1999;40(2):414–24. . 9950601

[46] Chang CH, Huang TL, Huang SP, Tsai RK. Neuroprotective effects of recombinant human granulocyte colony-stimulating factor (G-CSF) in a rat model of anterior ischemic optic neuropathy (rAION). Exp Eye Res. 2014;118:109–16. 10.1016/j.exer.2013.11.012 . 24316388

[47] Lindqvist N, Peinado-Ramonn P, Vidal-Sanz M, Hallbook F. GDNF, Ret, GFRalpha1 and 2 in the adult rat retino-tectal system after optic nerve transection. Exp Neurol. 2004;187(2):487–99. 10.1016/j.expneurol.2004.02.002 . 15144875

[48] Jehle T, Dimitriu C, Auer S, Knoth R, Vidal-Sanz M, Gozes I, et al The neuropeptide NAP provides neuroprotection against retinal ganglion cell damage after retinal ischemia and optic nerve crush. Graefes Arch Clin Exp Ophthalmol. 2008;246(9):1255–63. 10.1007/s00417-007-0746-7 . 18414890

[49] Wang R, Sun Q, Xia F, Chen Z, Wu J, Zhang Y, et al Methane rescues retinal ganglion cells and limits retinal mitochondrial dysfunction following optic nerve crush. Exp Eye Res. 2017;159:49–57. 10.1016/j.exer.2017.03.008 . 28336261

[50] Song WT, Zhang XY, Xia XB. Atoh7 promotes the differentiation of Muller cells-derived retinal stem cells into retinal ganglion cells in a rat model of glaucoma. Exp Biol Med (Maywood). 2015;240(5):682–90. 10.1177/1535370214560965 . 25710928 PMC4935277

[51] Hattar S, Kumar M, Park A, Tong P, Tung J, Yau KW, et al Central projections of melanopsin-expressing retinal ganglion cells in the mouse. Journal of Comparative Neurology. 2006;497(3):326–49. 10.1002/cne.20970 16736474 PMC2885916

[52] Li XT, Ren CR, Huang L, Lin B, Pu ML, Pickard GE, et al The Dorsal Raphe Nucleus Receives Afferents From Alpha-Like Retinal Ganglion Cells and Intrinsically Photosensitive Retinal Ganglion Cells in the Rat. Invest Ophth Vis Sci. 2015;56(13):8373–81. 10.1167/iovs.15-16614 26747768

[53] Ren CR, Pu ML, Cui Q, So KF. Dendritic Morphology of Caudal Periaqueductal Gray Projecting Retinal Ganglion Cells in Mongolian Gerbil (Meriones unguiculatus). Plos One. 2014;9(7). 10.1371/journal.pone.0103306 25054882 PMC4108400

[54] Conte WL, Kamishina H, Reep RL. Multiple neuroanatomical tract-tracing using fluorescent Alexa Fluor conjugates of cholera toxin subunit B in rats. Nat Protoc. 2009;4(8):1157–66. 10.1038/nprot.2009.93 19617887

[55] Duan X, Qiao M, Bei F, Kim IJ, He Z, Sanes JR. Subtype-specific regeneration of retinal ganglion cells following axotomy: effects of osteopontin and mTOR signaling. Neuron. 2015;85(6):1244–56. 10.1016/j.neuron.2015.02.017 . 25754821 PMC4391013

